# A linear B-cell epitope close to the furin cleavage site within the S1 domain of SARS-CoV-2 Spike protein discriminates the humoral immune response of nucleic acid- and protein-based vaccine cohorts

**DOI:** 10.3389/fimmu.2023.1192395

**Published:** 2023-05-05

**Authors:** Peter Lorenz, Felix Steinbeck, Franz Mai, Emil C. Reisinger, Brigitte Müller-Hilke

**Affiliations:** ^1^ Institute of Immunology, Rostock University Medical Center, Rostock, Germany; ^2^ Core Facility for Cell Sorting and Cell Analysis, Rostock University Medical Center, Rostock, Germany; ^3^ Division of Tropical Medicine and Infectious Diseases, Center of Internal Medicine II, Rostock University Medical Center, Rostock, Germany

**Keywords:** SARS-CoV-2, COVID-19, Spike protein, humoral immunity, antibody, linear epitope, peptide microarray, ELISA

## Abstract

**Background:**

Understanding the humoral immune response towards viral infection and vaccination is instrumental in developing therapeutic tools to fight and restrict the viral spread of global pandemics. Of particular interest are the specificity and breadth of antibody reactivity in order to pinpoint immune dominant epitopes that remain immutable in viral variants.

**Methods:**

We used profiling with peptides derived from the Spike surface glycoprotein of SARS-CoV-2 to compare the antibody reactivity landscapes between patients and different vaccine cohorts. Initial screening was done with peptide microarrays while detailed results and validation data were obtained using peptide ELISA.

**Results:**

Overall, antibody patterns turned out to be individually distinct. However, plasma samples of patients conspicuously recognized epitopes covering the fusion peptide region and the connector domain of Spike S2. Both regions are evolutionarily conserved and are targets of antibodies that were shown to inhibit viral infection. Among vaccinees, we discovered an invariant Spike region (amino acids 657-671) N-terminal to the furin cleavage site that elicited a significantly stronger antibody response in AZD1222- and BNT162b2- compared to NVX-CoV2373-vaccinees.

**Conclusions:**

Understanding the exact function of antibodies recognizing amino acid region 657-671 of SARS-CoV-2 Spike glycoprotein and why nucleic acid-based vaccines elicit different responses from protein-based ones will be helpful for future vaccine design.

## Introduction

The global pandemic “coronavirus disease 2019” (COVID-19) has been caused by the zoonotic severe acute respiratory syndrome coronavirus 2 (SARS-CoV-2). Its high socioeconomic impact is evidenced by a total of approximately 680 million confirmed infections as of March 2023, an estimated pandemic-related death toll of 6.88 million, and an unpredictable long-term post-COVID impact [https://www.arcgis.com/apps/dashboards/bda7594740fd40299423467b48e9ecf6 (1)].

To combat the pandemic, science, medicine, and industry have joined forces to rapidly develop safe vaccines that aimed to prevent severe disease and possibly restrict the propagation of the virus. Among the most used vaccines are those that are based on nucleic acids, either encoding the genetic information for SARS-CoV-2 Spike protein within a replication-deficient DNA adenoviral vector (e.g. vaccine AZD1222, Vaxzervria from Astra Zeneca (2);) or as stabilized mRNA packed within lipid nanoparticles (e.g. vaccine BNT162b2, Comirnaty from Pfizer/BioNTech (3); and mRNA-1273 from Moderna (4);). Later on, more classical protein-based vaccines such as NVX-CoV2373 (Nuvaxovid from Novavax (5);) were produced and authorized. The sequence of the SARS-CoV-2 Spike protein of all four vaccines mentioned above is derived from the wildtype Wuhan-Hu-1 virus (NCBI accession MN908947). However, unlike AZD1222, the three vaccines BNT162b2, mRNA-1273, and NVX-CoV2373 contain the double proline exchange of amino acids KV at positions 986 and 987 that stabilizes Spike in the so-called prefusion conformation, which is beneficial to raise neutralizing antibodies (6). Furthermore, three arginines within the furin cleavage site separating the S1 and S2 parts of Spike were mutated in the NVX-CoV2373 protein vaccine [so-called 3Q modification 679-NSPQQAQSVAS-689). All four vaccines were shown to be safe and effective (reviewed in (7, 8)].

Antibody epitope mapping enables the in-depth study of the humoral immune response towards SARS-CoV-2 antigens after both infection and vaccination (e.g (9–13).). Analyses of the resulting antibody landscapes provide essential insights into understanding and combating COVID-19: Antibody epitope patterns allow for the stratification of patients and may help distinguish groups with different pathophysiological backgrounds or clinical outcomes ( (12, 14). Knowing the exact epitopes that are recognized by antibodies can help to develop better and/or cheaper diagnostic assays, not only for specialized laboratories but also for point-of-care units. Patterns of immune reactivity may explain the lack of full protection against SARS-CoV-2 viral variants and serve as the basis for informed vaccine improvements. Moreover, antibodies that are tailor-made based on epitope mapping and that have high capacities for binding and neutralizing variants of concern (VOC) will yield substantial therapeutic potential.

While there is a large body of literature on Spike B cell epitopes related to infection by SARS-CoV-2 (e.g (11–13).; see 68 references at www.iedb.org for SARS-CoV-2 Spike protein Uniprot P0DTC2 as of February 2023), studies that compare antibody epitope profiles resulting from the different vaccines are still scarce. We, therefore, used peptide microarray and peptide-ELISA approaches to profile IgG-type antibody patterns against linear 15mer sequences in plasma samples of three vaccine groups. In detail, we compared AZD1222, BNT162b2, and NVX-CoV2373 vaccinated blood donors to COVID-19 patients and a pre-pandemic cohort. Our results show individual landscapes of immune reactivity without strong cohort-specific clustering. However, region 657-671 within the subdomain 2 of the SARS-CoV-2 Spike protein, just prior to the S1/S2 furin cleavage site, was discovered to distinguish the nucleic acid-based from the protein-based vaccine groups. Since these residues have not yet been mutated in variants, the sequence 657-671 should be considered when developing vaccines and therapeutics that prevent viral escape.

## Materials and methods

### Study participants and sample collection

The study was approved by the ethics committee of the Rostock University Medical Center under file number A 2020-0086. All donors filed their written informed consent for participation. Apart from sex, age, sampling dates, SARS-CoV-2-related PCR, and serological data, no other clinical parameters or comorbidities were recorded. COVID-19 patient and pre-COVID cohorts were recruited between September 2020 and January 2021, before the advent of routine vaccination for the general population (15). In detail, pre-COVIDs were recruited from the local test center that they visited for the exclusion of COVID-19. Inclusion in our study required a negative PCR result for SARS-CoV-2. Patients with COVID-19 were recruited from the Division of Tropical Medicine and Infectious Diseases at Rostock University Medical Center within the first two weeks of infection. Infection was confirmed by a SARS-CoV-2-specific diagnostic PCR. Blood samples were collected *via* venipuncture in the presence of EDTA as an anti-coagulant and plasma was harvested after centrifugation. Plasma aliquots were stored at -80°C. Subjects for the vaccine cohorts were enrolled from clinical, scientific, and administrative staff members of the Rostock University Medical Center (AZD1222, BNT162b2, and NVX-CoV2373) and from the local population at municipal vaccination centers (NVX-CoV2373). Vaccinations followed the recommendations of the German Standing Committee on Vaccination (STIKO). The AZD1222 “A” cohort received two doses of AZD1222 at a mean interval of 12 weeks. Boost immunizations with an mRNA-based vaccine (either BNT162b2 or mRNA-1273) were administered at a mean interval of 39 weeks after priming. The BNT162b2 “B” cohort received two doses of BNT162b2 at a mean interval of 29 days. Boost immunizations with either BNT162b2 or mRNA.1273 were administered at a mean interval of 44 weeks after priming. The “N” cohort received two doses of NVX-CoV2373 at the recommended interval of 21 days. Inclusion into any of the vaccine groups at any time point required negative test results for anti-nucleocapsid antibodies. Blood samples were collected between July 2021 and October 2022.

### Evaluation of SARS-CoV-2 Spike, nucleocapsid, and neutralizing antibody reactivity in plasma samples

Antibodies of type IgG against SARS-CoV-2 Spike RBD domain or nucleocapsid protein were quantified using commercial anti-SARS-CoV-2S and anti-SARS-CoV-2N Elecsys^®^ immunoassays (Roche Diagnostics, Mannheim, Germany). The assays were done by the central laboratory of the Institute of Clinical Chemistry and Laboratory Medicine of the Rostock University Medical Center. Antibody reactivity is referred to as the international WHO standard for SARS-CoV-2 immunoglobulins and is expressed as binding antibody units (BAU) (16). A commercial surrogate virus neutralization assay (sVNT, GeneScript) was used to determine the neutralization capacities of plasma samples as described previously (17).

### Peptide microarray

We employed a commercially available peptide microarray slide (JPT Peptide Technologies GmbH, product RT-WCPV-S-V04-2) carrying 21 identical microarrays that include 318 peptides derived from SARS-CoV-2 Wuhan-Hu-1 wildtype Spike protein sequences (P0DTC2; Uniprot release 2021_02). The annotated peptide content of a microarray can be found as part of the data record in **Supplementary Table S1**. The peptides are 15mers with 11 amino acid overlaps and are covalently attached to the glass slide *via* an N-terminal spacer. Because of technical reasons, they carry an additional glycinate residue at their C-terminus. A 96-well frame (JPT Peptide Technologies GmbH) that carries up to four slides separates the subarrays into individual wells. To get replicate measurements, each sample was applied to three subarrays. Subarrays that were only probed with secondary antibodies served as a baseline for later quantitation. Incubations of the microarray slide were done on a plate shaker (Thermo Fisher) at 30°C. The staining workflow commenced with the blocking step using SuperBlock/TBS (Thermo Fisher) with 0.5% Tween 20 for 1 hour and two rinses with washing buffer (TBS, 50mM TRIS/HCl pH 7.4, 150mM NaCl plus 0.1% Tween 20). Next, the subarrays were probed with plasma pools of different sample groups diluted in the blocking buffer for 2 hours. Each pool was made up of 10 plasma samples in which each sample was diluted 1:100. Subarrays were rinsed five times with washing buffer and then incubated for 1 hour with a secondary antibody Fab preparation of goat anti-human IgG conjugated with Alexa Fluor 647 (Thermo Fisher Z25408), diluted to 0.8 µg/ml in blocking buffer. After five rinses in washing buffer and two rinses in distilled water, the microarray slide was dried by brief centrifugation and then scanned in a fluorescence scanner (Agilent G2505C) at 5µm per pixel resolution and with 16-bit sample depth. Image analysis, including spot detection, outlier correction, and filtering for reactive peptides over background, was done as described in detail in a previous study (18). Briefly, GenePix Pro version 6.1 (Molecular Devices) was used to determine the median foreground fluorescence of each spot. Then, the fluorescence intensity of the three replicates was averaged. The list of peptides with specific signals over background was derived by using the MAID approach with a signal threshold of 400 that considers the criteria signal intensity as well as fold change to the buffer control. The selection of peptides for further study was done manually using the following criteria: We first built groups of all peptides with signals over background according to their reactivity with plasma samples from the cohorts. These included groups “vaccinees and patients”, “vaccinees NOT patients”, “AZD1222 only” and “BNT162b2 only”, “patient only”, “pre-pandemic only” and “pre-pandemic & patient”. We then removed all peptides present only in Spike protein sequences from SARS-CoV-2 variants. Within each of the peptide reactivity groups, we chose the peptides with the highest reactivity. This could be the maximum for single cohort groups or the sum of signals when more than one cohort contributed to a selection group. When peptides were overlapping, we selected the one with the highest signal.

### Peptide ELISA

Peptides of 15 amino acid length with N-terminal biotin, spacer, and an additional glycinate at their C-terminus were obtained from a commercial source (JPT Peptide Technologies GmbH, BioTides™). Prior to first use, peptides were dissolved at 0.2mM in dimethylsulfoxide and stored at -80°C. Further dilutions were done in TBS (50mM TRIS/HCl, 150mM NaCl). Polystyrene 96-well plates (NUNC Maxisorp, Thermo Fisher) were coated with 100µl of a solution of 5µg/ml Neutravidin (Thermo Fisher) in 0.1M carbonate buffer pH 9.6 at 4°C for 18 hours. After rinsing twice with TBS, wells were loaded with 50 pmoles per well biotinylated peptide in 100µl TBS for 1 hour at 37°C. Wells were rinsed twice with PBS and then blocked with 300µl Superblock/TBS (Thermo Fisher) at 37°C for 1 hour, followed by another two rinses with TBS. The plasma samples were diluted 1:200 in antibody dilution buffer (Superblock/TBS plus 0.5% Tween20 and 10µg/ml Neutravidin, the latter to pre-adsorb potential Neutravidin-reacting immunoglobulins) and incubated in 100µl volumes per well at room temperature for 2 hours. Each well was rinsed once, washed twice (5 min incubations), and rinsed again with washing buffer (TBS, 0.1% bovine serum albumin, 0.5% Tween 20). Bound primary antibodies from the plasma samples were probed with goat anti-human IgG antibodies conjugated to peroxidase (Jackson Dianova 109-035-088) at room temperature for 1 hour. Following the washing steps as described after the plasma sample incubation, the substrate reaction with TMB (BioLegend #421101) was initiated until the OD at 620nm (blue) of a chosen peptide/plasma sample used on each ELISA plate reached 0.5, usually approximately 20 minutes. Then the reaction was stopped with 2N sulfuric acid and the OD was read at 450nm (yellow). Plasma-specific peptide reactivity was calculated by subtracting the OD 450nm of a well without peptide from the raw OD 450nm of the well with peptide probed with the same plasma sample. If this difference was negative, the value was set to zero. Preliminary testing in independent triplicate experiments and using only a selection of nine peptides and 19 plasma samples resulted in overall robust ELISA data with high Pearson correlation (median values 0.94; minimum 0.73). For this reason, we decided to determine the whole dataset by single measurements.

### Computational tools, statistics, and figure preparation

We used packages of the R software environment (www.r-project.org) as well as the methods and tools implemented in JASP v 0.16.4 (Sept 29, 2022; https://jasp-stats.org) to draw data illustrations and to statistically evaluate them as indicated in the figure legends and in the text. Initial graphs were imported into Corel Draw X8 for post-processing to get the final figure layout and to add labeling. The data of multiple groups were compared with the Kruskal-Wallis test and, if a significance level of p < 0.05 was reached, the data was subjected to Dunn’s *post-hoc* analysis with Benjamini-Hochberg correction for multiple testing. The longitudinal series were evaluated using the Friedman test followed by a Conover *post-hoc* analysis when initial p-values were smaller than 0.05. Correlations of anti-peptide signal intensities with values of the commercial anti-RBD ELISA as well as the neutralization assay are based on Spearman’s rank correlation coefficient (rho and p-value; https://www.wessa.net/rwasp_spearman.wasp). To compile known B-cell epitopes, we searched the Immune Epitope Database and Analysis Resource (www.iedb.org (19); accessed on 2023-01-23) for hits within Spike protein of SARS-CoV-2 Wuhan-Hu-1 (Uniprot P0DTC2). In particular, we focused on sequence S-657 using the search options “linear peptide”, “substring” “B cell assay: any” and “Host: human”. In addition, we screened publications with the subject “B-cell epitope mapping” of SARS-CoV-2 Spike protein and extracted the reported immunodominant hits. The display of 3D protein structures was done with the help of PyMol 2.5.0 (Schrödinger, LLC).

## Results

### Landscapes of Spike peptide reactivity indicate overlapping as well as individual profiles

In the first approach, we compared linear B-cell epitopes recognized by IgG-type antibodies in the plasma of SARS-CoV2 vaccinees who received either vector-based AZD1222 (n=20) or mRNA-based BNT162b2 (n=20). The samples from the vaccinated cohorts were compared to a control group of 10 pre-COVID plasma samples and 10 samples from COVID-19 patients with active disease. The groups were matched for age and sex (**Table 1**). To obtain an overview of the various reactivity profiles, we used pools of plasma representing the sample groups and stained a commercially available peptide microarray carrying overlapping 15mer peptides that cover the whole Spike protein sequence from wildtype Wuhan-Hu-1 (**Supplementary Figure S1**). The quantitative evaluation of the peptide microarray images revealed that 115 out of 318 wildtype peptides were specifically recognized by at least one of our plasma pools. The summary of all peptides and heatmaps illustrating the reactivity landscape are documented in **Supplementary Table S1** and **Supplementary Figure S2**, respectively.

**Table 1:** Demographics of the study participants.

Cohort	pre-COV	AZD1222	BNT162b2	NVX-CoV2373	COV
**n**	10	20	20	11	10
Age [mean ± SD]*	45.7 ± 19.4	41.1 ± 13.4	38.3 ± 12.3	44.5 ± 12.5	41.1 ± 17.2
**Sex** **[male/female]^#^**	4/6	10/10	5/15	5/6	4/6

* p = 0.733 (Kruskal-Wallis).

^#^ p = 0.583 (Chi-squared test).

**Figure 1:**
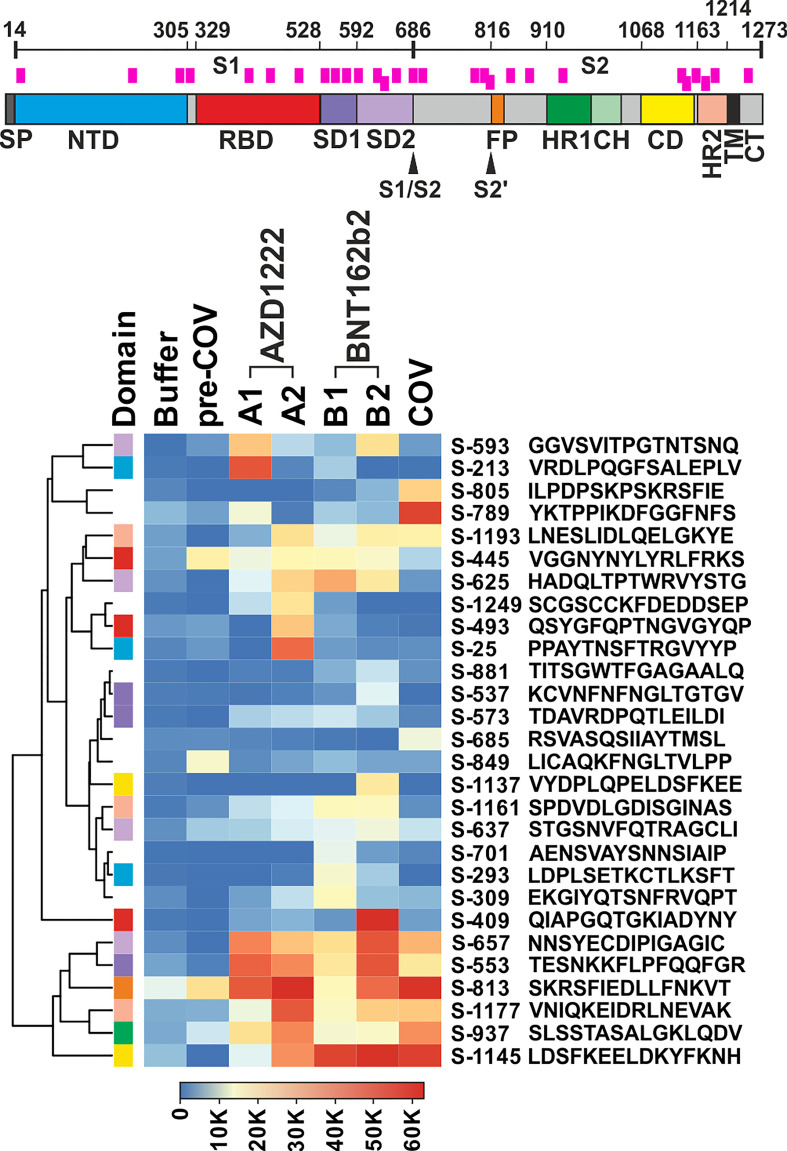
Plasma from vaccine groups and COVID-19 patients revealed overlapping and individual reactivity profiles. Top: Schematic of the SARS-CoV-2 Spike protein. Domain annotation of Uniprot P0DTC2 according to (20). Peptides shown in the heatmap are indicated as pink bars. SP, signal peptide; NTD, N-terminal domain of part S1; RBD, receptor binding domain; SD1, SD2, subdomains 1 and 2; S1/S2, furin cleavage site; S2’= S2 sub-cleavage site; FP, fusion peptide; HR1, HR2, heptad repeats 1 and 2; CH, central helix; CD, connector domain; TM, transmembrane domain; and CT, cytoplasmic tail. Bottom: Heatmap of selected peptides after quantitative analysis and filtering of peptide microarray data obtained with pools of plasma samples from the different cohorts as described in A. Names of the peptides derived from the antigen (“S” for Spike) and their first amino acid numbered according to position in the full-length protein. Peptides are further annotated by the respective protein domains they derive from (“Domain”, see Spike drawing.). Hierarchical clustering of the peptides used Euclidean distance and complete linkage.

We filtered the data for hits in SARS-CoV-2 Spike wildtype peptides that showed either high reactivity or potentially interesting patterns. Among the latter, we considered reacting with plasma from patients and/or vaccinees (see Methods for details). The curation of all signals resulted in the selection of 28 peptides for further analyses (**Figure 1**). Note that peptide S-813 covering part of the “fusion peptide” around the S2’ sub-cleavage site did not pass our background selection filter. However, since we found very high reactivity among all vaccinees and patients cohorts and because this region is important for virus entry into the host cell (21), we decided to include this peptide in our further study. Of note, the 28 selected peptides are distributed throughout the Spike protein sequence, including its receptor binding domain (RBD). Nevertheless, **Figure 1** shows a more dense coverage of the Spike S1 SD1 and SD2 domains as well as of CD and HR2 domains within the S2 part.

The overall signal patterns of the 28 selected as well as all reactive peptides (**Figure 1**; **Supplementary Figure S2B**) showed many overlaps between the sample cohorts. For example, six of the selected peptides (S-553, S-657, S-813, S-937, S-1145, and S-1177) displayed very high antibody signals for both vaccinees and patients. Others showed a more focused distribution with prevalent reactivity only for one cohort, e.g.S-213 for AZD1222, S-409 for BNT162b2, or S-789 for the patient samples. A formal analysis of the cohorts with respect to all reactive peptides corroborated overlapping as well as cohort-specific reactivity profiles (see **Supplementary Figure S3**; Venn diagram). Since peptide-specific signals were derived from pools of 10 plasma samples each, which in turn may contain polyclonal and polyspecific antibodies, the recorded signals reflect the summation of the reactivity of many antibodies.

### Linear B cell epitopes around SD1, the fusion peptide, and the connector domain of S2 distinguish COVID-19 patients from vaccinees

We next set out to resolve the sample pools. To that end, we used ELISA and determined the reactivity of each individual plasma against the 28 selected peptides. For this series of experiments, we included a total of 71 samples: 20 BNT162b2 vaccinees, 20 AZD1222 vaccinees, 10 COVID-19 patients with active disease, 10 pre-COVID samples, and an additional 11 samples representing NVX-CoV2373 vaccinees (**Supplementary Table S2**). Against expectation, one of the pre-COVID plasma samples, pre-COV5, displayed very high reactivities towards 11 peptides and was therefore defined as an outlier and excluded from further analyses (**Supplementary Table S2**).

**Figure 2:**
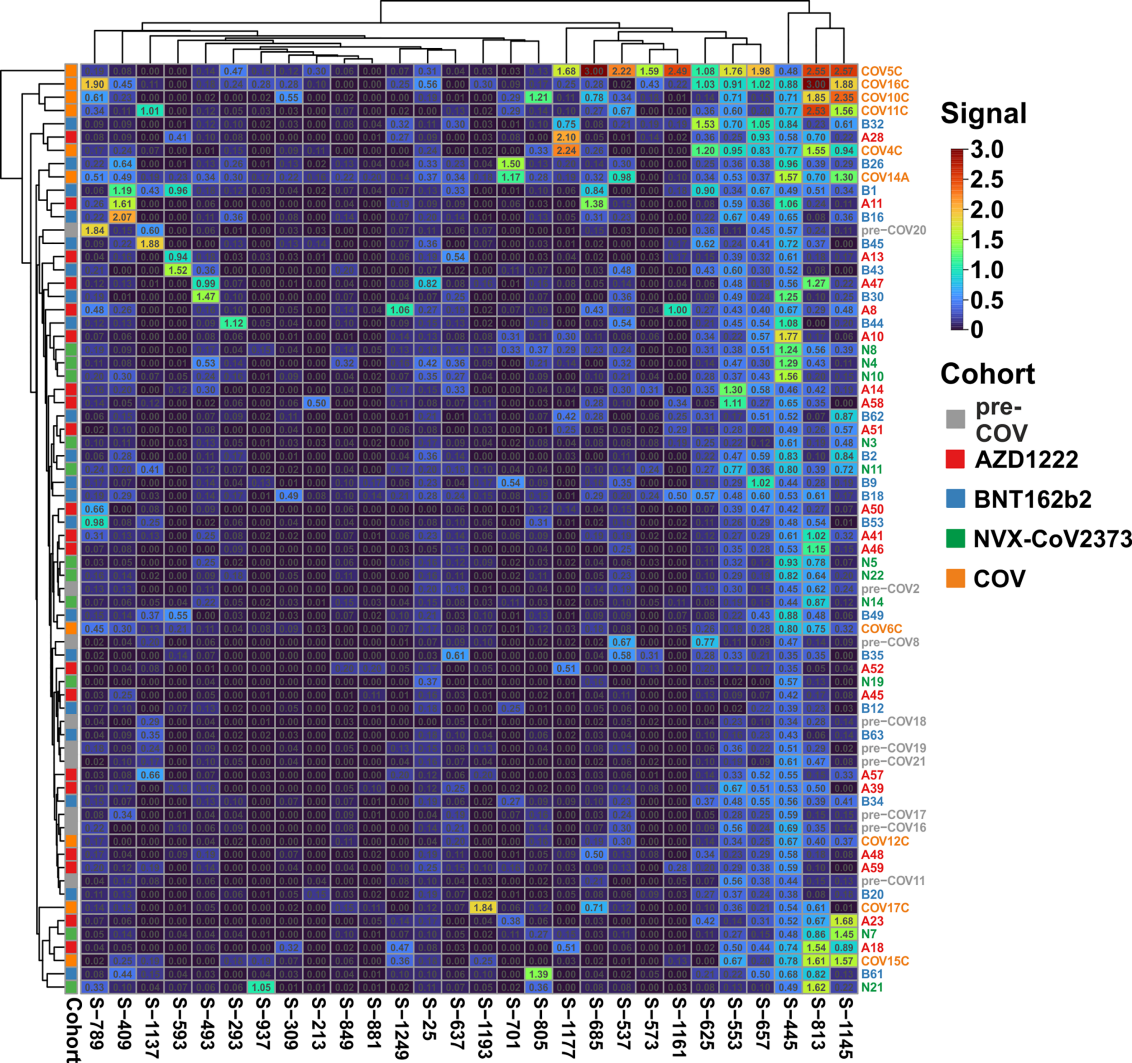
The antibody reactivity landscape against Spike-derived peptides indicated robust humoral immunity for the patient and vaccine cohorts Overview heatmap of background-corrected OD450nm values for 28 individual peptides and plasma samples of the COVID-19 patient cohort (COV; n = 10), three vaccine groups (AZD1222, n = 20; BNT162b2, n = 20; and NVX-CoV2373, n = 11) in comparison to pre-COVID-19 samples (pre-COV, n = 9) using peptide-ELISA. Samples are color-coded to the left of the heatmap according to cohort (legend to the right). Clustering of both samples and peptides, with Euclidean distance and complete linkage.

**Figure 3:**
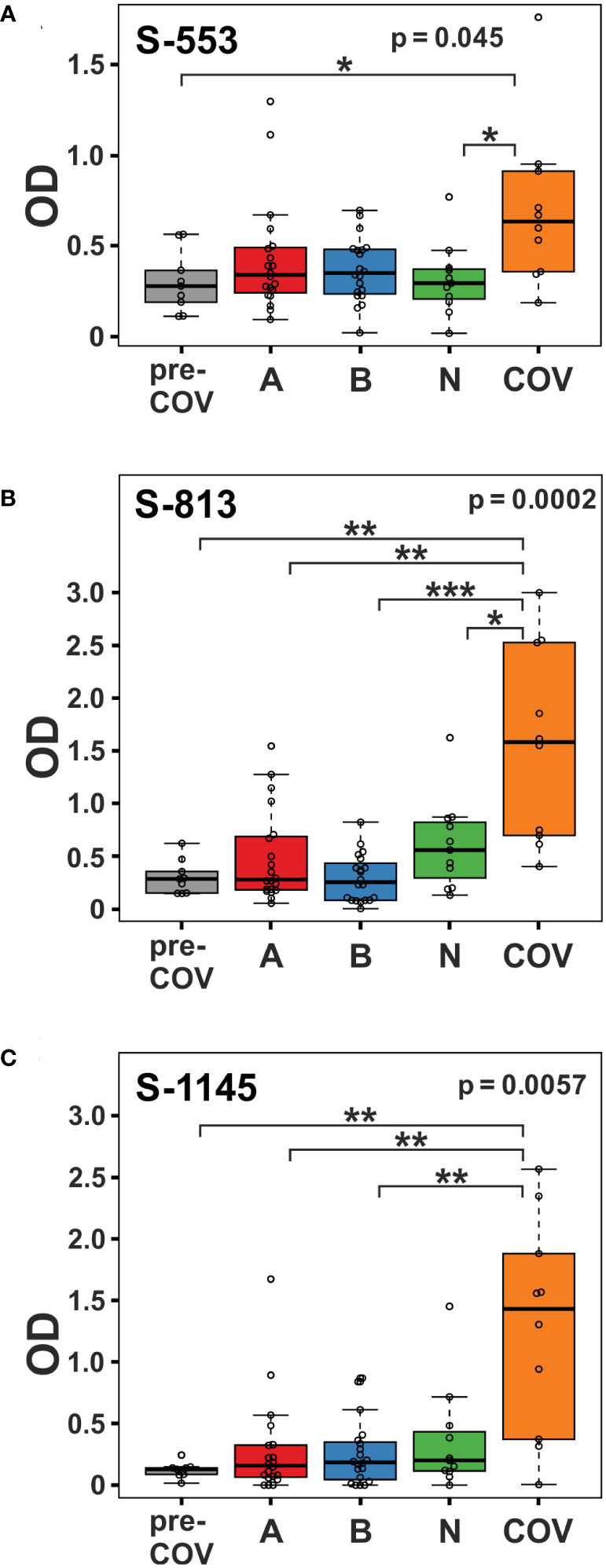
Viral infection in COVID-19 patients elicits stronger immune reactivity towards peptides S-553, S-813, and S-1145 than vaccination. Reactivity of plasma samples from the pre-pandemic group (pre-COV), the three vaccinee cohorts (A = AZD1222; B = BNT162b2; and N = NVX-CoV2373), and the patients with peptide S-553 **(A)**, S-813 **(B)**, and S-1145 **(C)** as measured by peptide ELISA. Signals represent the background-corrected optical density values (OD). Significance levels for the comparison of the cohorts shown on the top right of each plot (Kruskal-Wallis) with individual group *post-hoc* comparisons on brackets (Dunn’s test; *p < 0.05; **p< 0.01; ***p< 0.001).

Clustering of the ELISA results showed high signals beyond OD 2.0 (orange and red tiles in the heatmap) in five of the 10 samples from patients with active disease, whereas in the vaccine and pre-COV cohorts, only one sample each from groups AZD1222 and BNT162b2 reached this level (**Figure 2**). In particular, sample COV5C stood out with the highest reactivities for seven out of the 28 peptides (S-537, S-553, S-573, S-657, S-685, S-1145, and S-1161) In total, we found eight peptides with significant differences between groups (Kruskal-Wallis p < 0.05 and Dunn’s *post hoc* values p < 0.05) with prominent reactivities against S-553, S-813, and S-1145 (median signals > 0.5 OD; maximum value >1 OD) among patients (**Figure 3**). These peptides derive from the subdomain SD1 region of Spike S1 (S-553), the fusion peptide (S-813), and the connector domain CD of Spike S2 (S-1145), respectively. The statistical *post-hoc* analyses confirmed robust differences between patients and NVX-CoV2373 vaccinees (S-553), between patients and all vaccination groups (S-813), and between patients and nucleic acid-based vaccine cohorts (S-1145) (**Figure 3**).

### A linear B-cell epitope close to the furin cleavage site of Spike S1 distinguishes the protein- from the nucleic acid-based vaccine groups

The heatmap in **Figure 2** illustrates high variability and sample-specific reactivity patterns rather than cohort-specific ones. When focusing on the vaccine cohorts, there was only one small vaccine-specific cluster consisting of three NVX-CoV2373 samples (N8, N4, and N10). Other than that, most peptides reacted selectively with a small subset of plasma samples. With respect to signal intensities, nine peptides displayed signals above 1 OD, while eight never reached values over 0.8 OD. Interestingly, peptide S-445, featuring the highest number of recognitions with many values above 1 OD (7/51 of all vaccine cohort samples; three of them from the NVX-CoV2373 cohort), is part of the Spike RBD. Altogether, the humoral response to vaccination turned out to be individually diverse yet relatively limited in its breadth with respect to the recognition of the 28 selected peptides. However, six peptides stood out because of their differences between cohorts (Kruskal-Wallis p-value < 0.05; see **Supplementary Table S3**). At closer inspection though, five of these, S-293, S-309, S-805, S-849, and S-937 yielded very low signal intensities and upon *post-hoc* tests, three of them (S-805, S849, and S-937) failed to reveal group-specific differences (Dunn’s test p > 0.05).

**Figure 4:**
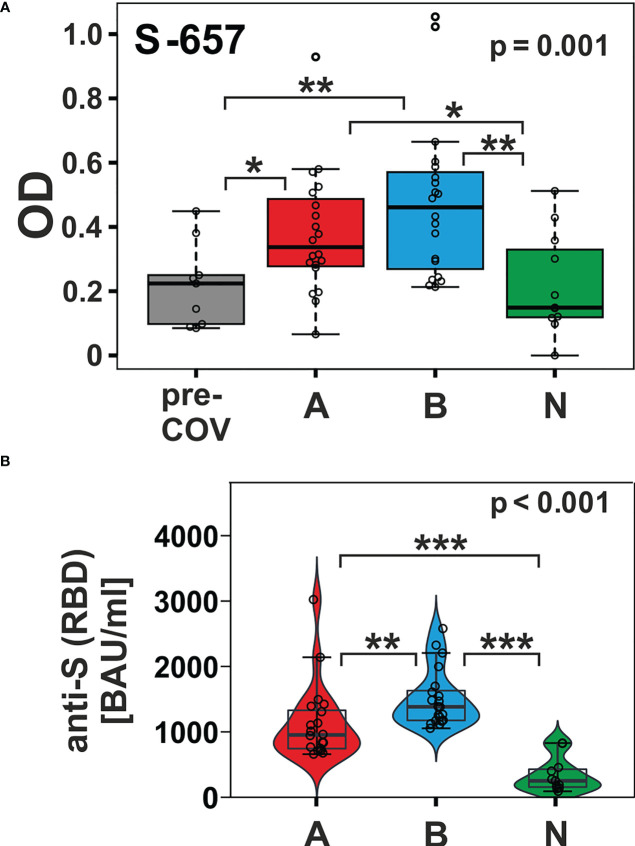
The humoral immune response towards peptide S-657 distinguished subjects immunized with nucleic acid-based vaccines from those that received the protein-based vaccine and from pre-COVID-19 samples. **(A)** Reactivity of plasma samples from three vaccinee cohorts (A = AZD1222; B = BNT162b2; and N = NVX-CoV2373) and the pre-pandemic group (pre-COV) with peptide S-657 as measured by peptide ELISA. Signals represent the background-corrected optical density values (OD). Data were statistically evaluated for changes between sample groups. **(B)** Measurement of anti-S (RBD = receptor binding domain) antibody levels using a diagnostic sandwich immunoassay. Signals standardized as “BAU”= “binding antibody units” (WHO standard). Significance levels for the comparison of the cohorts shown on the top right of each plot (Kruskal-Wallis) with individual group *post-hoc* comparisons on brackets (Dunn’s test; *p < 0.05; **p< 0.01; ***p< 0.001).

In contrast, peptide S-657 exhibited robust signals that distinguished the AZD1222 and BNT162b2 vaccinees not only from the pre-pandemic control group but also from the NVX-CoV2373 vaccinees (**Figure 4A**; Dunn’s *post hoc* test p-values < 0.05). Though the BNT162b2 plasma samples exhibited a trend towards higher median values (0.461 vs. 0.337), the difference to the AZD1222 group was not statistically significant (Dunn’s *post hoc* p-value = 0.278). The differences between AZD1222 and BNT162b2 on the one hand and NVX-CoV2373 on the other were also apparent for Spike RBD recognition as measured by the diagnostic immunoassay (**Figure 4B**). This latter finding suggested that the differences in the extent of the humoral response between vaccine groups were not limited to S-657. As opposed to our linear peptide ELISA, the diagnostic anti-SARS-CoV-2 immunoassay uses a recombinant protein representing the whole RBD as antigen and can therefore detect antibodies towards discontinuous epitopes. In summary, our approach yielded the linear peptide S-657 – located close to the S1/S2 furin cleavage site - that elicited antibody responses following vaccination with AZD1222 and BNT162b2, but significantly less so after NVX-CoV2373 vaccination.

### Linear B-cell epitope reactivity is strongest after booster vaccination with longitudinal responses varying among individuals

Access to plasma samples from vaccine groups AZD1222 and BNT162b2, who received a third, i.e. booster immunization, allowed us to look at the dynamics of the response to selected linear B-cell epitopes. We compared the time point 6 months (T1) to 12 months (T2, i.e. 3 months after the booster) and 18 months (T3, 9 months after the booster) after primary immunization. Of note, AZD1222 participants were vaccinated following a heterologous regimen, since boosters consisted of mRNA vaccines (n=7 BNT162b2 and n=1 mRNA1273 from Moderna). In addition, two vaccinees from the BNT162b2 cohort received a second booster at 15 months.

**Figure 5:**
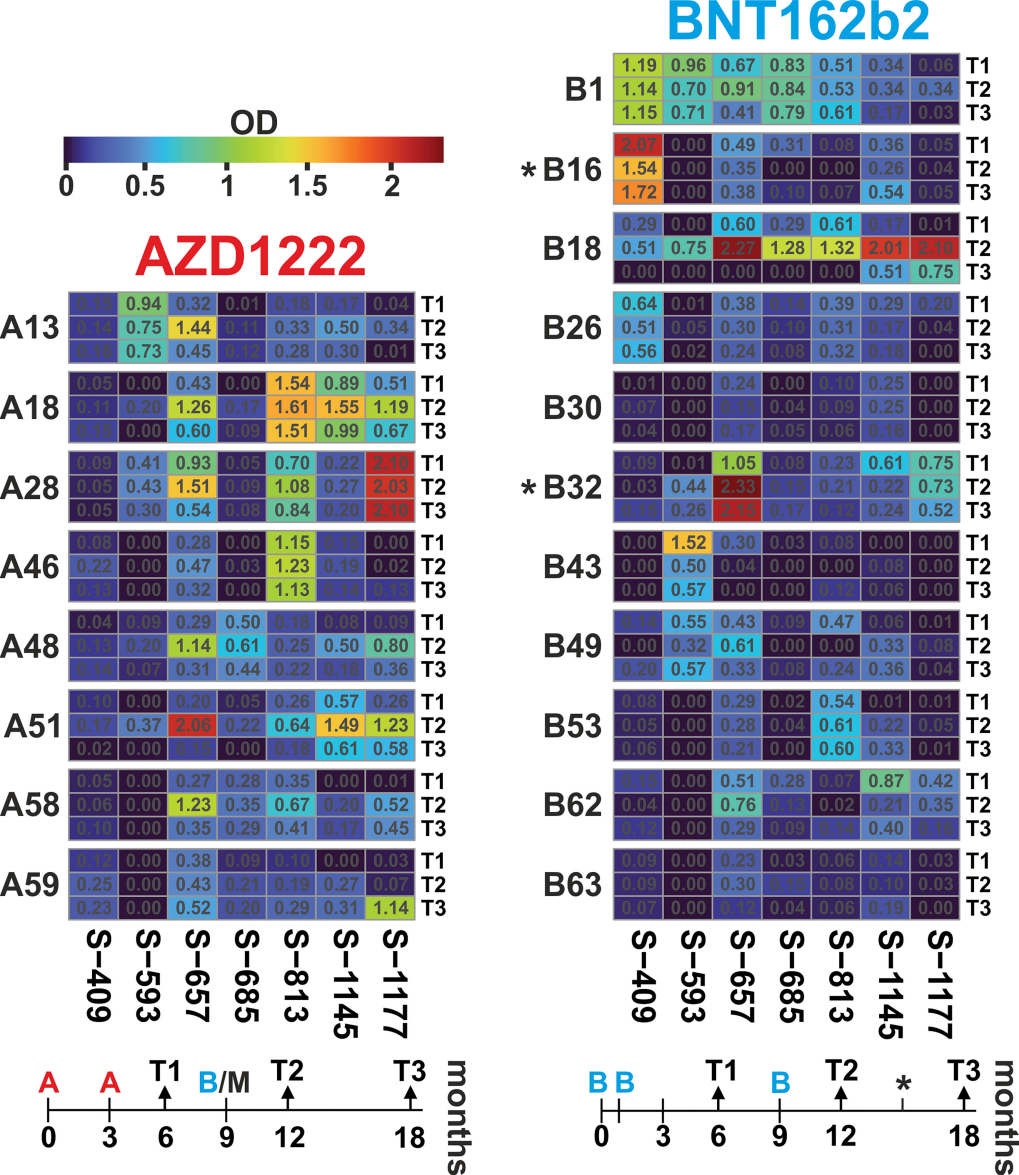
Antibody dynamics of the AZD1222 and BNT162b2 vaccine groups showed individual responses, with the highest reactivity predominantly three months after the booster immunization. Heatmaps of peptide-ELISA data (background-corrected OD450nm) obtained for the indicated vaccinees (A = AZD1222 and B = BNT162b2) at time points T1, T2, and T3 (6, 12, and 18 months after initial immunization). The temporal relation of sampling and immunization schemes is depicted below each heatmap. The time of immunization is indicated with A and B for the two vaccines, respectively. Note that all donors of the AZD1222 group received a booster injection with an mRNA vaccine, usually, BNT162b2 (in one case, mRNA1273 from Moderna), and two donors from the BNT162b2 cohort received a fourth injection at about 15 months (indicated by an asterisk).

For longitudinal analyses, we focused on seven peptides that showed either group-specific differences or high individual responses. The resulting profiles over the three-time points showed individual time courses (**Figure 5**; data in **Supplementary Table S4**). Most frequently, ELISA signals increased from T1 to T2 and waned again towards T3 with overall robust reactivity. Examples for this scenario are AZD1222 cohort members A18 and A51 or BNT162b2 vaccinee B18 (all for the three peptides S-657, S-1145, and S-1177). The statistical analysis stated that within the AZD1222 cohort, six of seven peptides had the highest response at time point T2 (Friedman test p < 0.05; exact values given in **Supplementary Table S5**). The same was true for two peptides (S-657 and S-1177) among the BNT162b2 vaccinated cohort. In another scenario, high ELISA signals remained elevated over the three-time points, as seen in donors A28 (S-1177), A46 (S-813), or B1 (S-409). However, there was also a scenario where initial reactivity was not boosted, e.g. cases A13, B1, and B43 (all for peptide S-593). Looking for differences between the AZD1222 and BNT162b2 vaccine cohorts, we found significantly higher reactivities for the AZD1222 group and peptides S-813 (at T2 as well as T3) and S-1177 (T3; Mann-Whitney test with p < 0.05; **Supplementary Table S5**). In summary, vaccinees benefited from booster vaccines in terms of increased antibody reactivities against various epitopes.

### Increased antibody reactivities to peptide S-657 after booster immunization correlated with antibody levels against Spike(RBD) protein and neutralizing capacities

**Figure 6:**
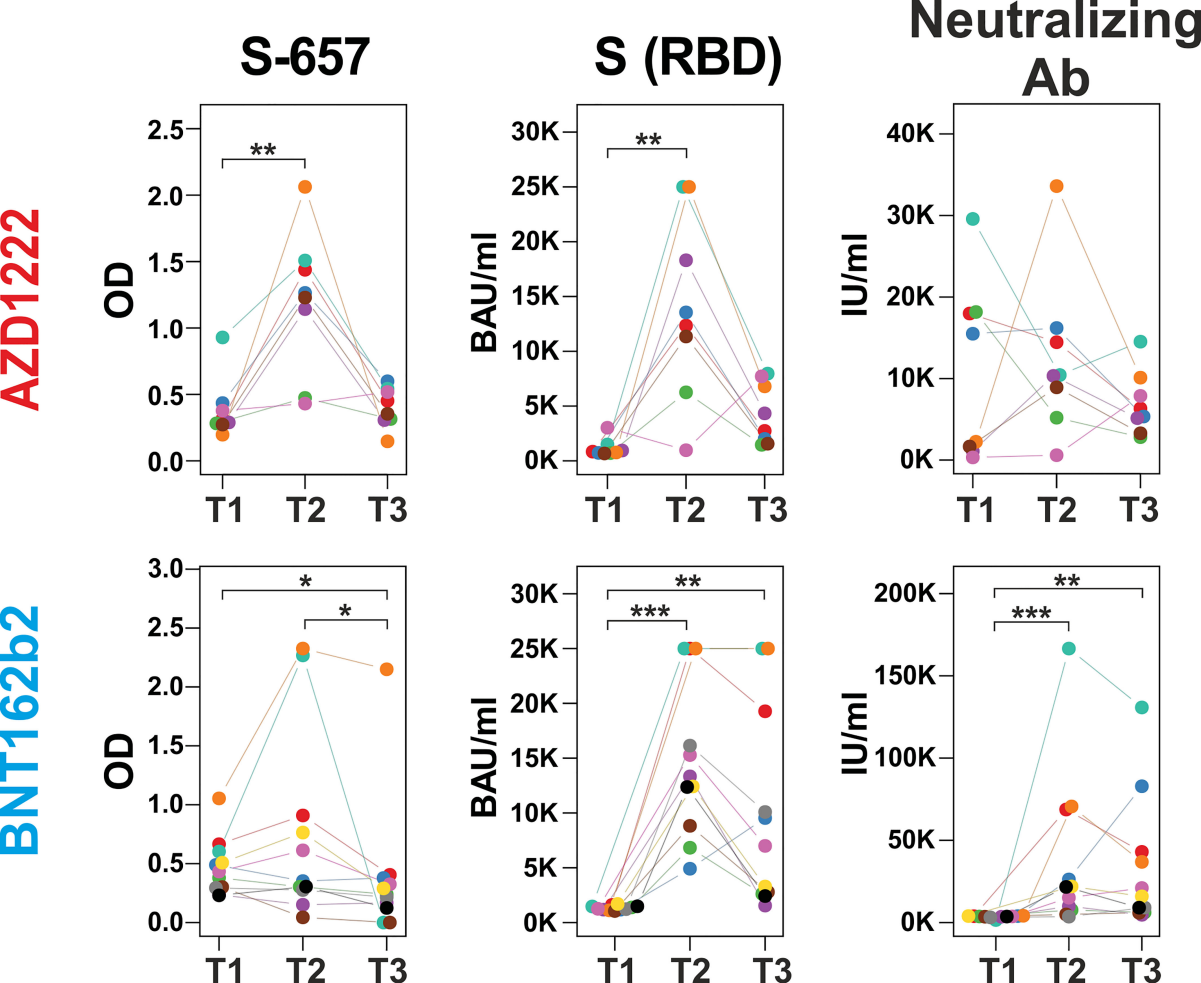
Increase of peptide S-657 reactivity three months after the booster immunization largely paralleled S(RBD) and neutralizing antibody levels Courses of antibody-derived signals from time points T1 (6 months), T2 (12 months) to T3 (18 months) against peptide S-657, S(RBD) protein and with neutralizing activity as measured with peptide ELISA (background-corrected OD 450), diagnostic ELISA (BAU = binding antibody units) and surrogate neutralization test (IU = international units), respectively. Samples of the same participant are color-coded. Each longitudinal series within an assay was statistically evaluated using a Friedman test. If p was smaller than 0.05, individual time points were compared using a Connover *post-hoc* test (*p < 0.05; **p < 0.01; ***p < 0.001).

In order to evaluate whether changes in linear peptide reactivity paralleled the more general anti-Spike(RBD) response, we focused again on peptide S-657 and compared its reactivity to anti-RBD titers and neutralizing capacities. Indeed, we found significant correlations for the individual donors between linear peptide and S(RBD) reactivity and between linear peptide reactivity and neutralizing capacity for the time point T2 at 12 months (**Figure 6**; **Supplementary Table S5**). Spearman correlations were at ρ > 0.8 and significance level p < 0.012 for the AZD1222 cohort for both correlations. For the BNT162b2 plasma samples, we determined ρ = 0.6 at p = 0.048 for the peptide to S(RBD) antibody value correlation and ρ = 0.9 with p < 0.001 for the comparison between peptide reactivity and neutralization capacity. In summary, the linear S-657 peptide response appeared as a surrogate for both anti-S(RBD) binding antibody units (BAU) and international units (IU) of neutralizing capacity.

## Discussion

We aimed to discriminate antibody binding patterns in different vaccine and SARS-CoV-2 patient cohorts. Including vaccinees who received either AZD1222, BNT162b2, or NVX-CoV2373, we found two important results: i) the linear peptide S-657, covering amino acids 657-671 of the Spike protein, discriminated nucleic acid- from protein-based vaccine cohorts and ii) peptides S-553, S-813, and S-1145 discriminated patients from vaccinees.

**Figure 7:**
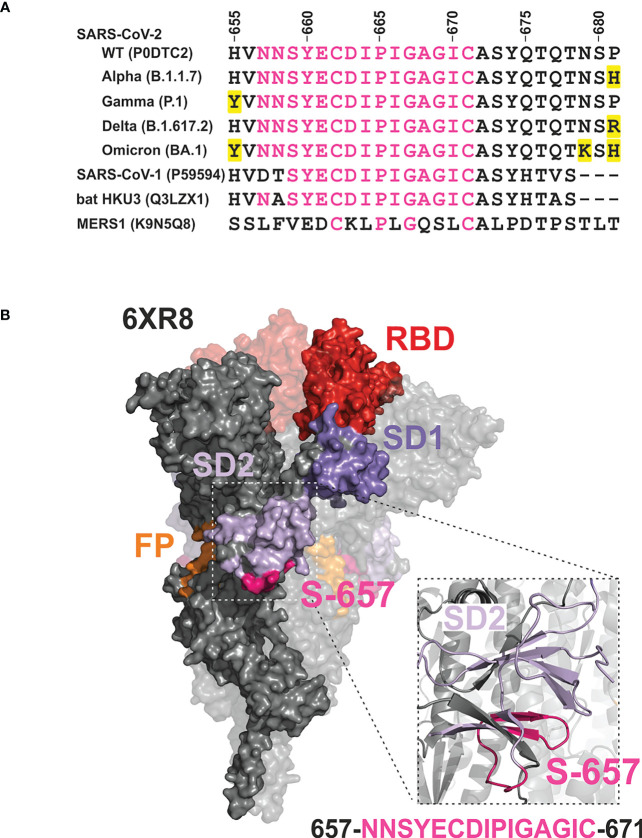
Mapping of the phylogenetically conserved sequence of peptide S-657 on a 3D cryo-EM structure of the Wuhan-Hu-1 wildtype Spike trimer implicates partial surface localization and antibody accessibility **(A)** Alignment of S-657 peptide of SARS-CoV-2 Spike protein (WT = wildtype) with related sequences from betacoronavirus family members (two from human and one from a bat as host; identifiers from Uniprot). The alignment is extended to contain amino acids 655-681 to include mutations (highlighted in yellow) in SARS-CoV-2 variants of concern nearby. However, these amino acid changes are not part of epitope S-657. Amino acids that are identical to those in S-657 are colored pink. **(B)** Surface representation of Spike trimer (PDB 6XR8) with one highlighted protomer and close-up cartoon view of the peptide S-657 region within SD2. Domains RBD (red), SD1 (darker purple), SD2 (lighter purple; colored as in **Figure 1**), and peptide sequence 657-671 (pink).

Information on the humoral immune response of vaccinees against sequences overlapping S-657 is very limited. One study found substantial reactivity for nine out of 14 BNT162b2 cases using a peptide microarray (9) while in a second study, the BNT162b2 cohort (25 subjects) was inconspicuous (10). A third investigation defined a large 151 amino acid segment including S-657 to be immunoreactive for BNT162b2-vaccinated donors. However, given the large antigen size, reactivity cannot be solely attributed to S-657 (22). In contrast to vaccinees, profiling of COVID-19 patients and/or convalescents evinced antibody binding to the S-657 sequence region in many cases (see **Supplementary Table S6**). The stretch of amino acids of S-657 covers a region within SD1 that has not yet been found mutated in variants of interest/concern. The closest amino acid exchanges found are H655Y, N679K, and P681H/P681R in variants Gamma, Omicron, or Alpha/Delta, respectively (**Figure 7A**). This sequence stability could indicate the importance of viral function. Likewise, mutations in this region might not confer a sufficient evolutionary advantage to transmit into viral isolates. Of note, the S-657 sequence of SARS-CoV-2 is highly homologous to Spike residues of other members of the betacoronavirus family, in particular SARS-CoV-1 and bat coronavirus (13 and 14 identical residues (11, 13);, see **Figure 7A**), supporting functional relevance.

On its C-terminal side, peptide S-657 is flanked by amino acids R685 and S686 that define the S1/S2 junction and a furin cleavage site. This polybasic furin recognition site has been shown to be important for efficient virus propagation (23, 24) and variants of concern carrying mutations at this position obtained higher transmissibility (reviewed in (25)). Mapping to a 3D structure of the trimeric Wuhan-Hu-1 wild-type Spike protein (26) revealed that the S-657 sequence consists of a larger and smaller loop between short beta-sheet secondary structures (**Figure 7B**). The overall topology suggests that a portion of the segment is exposed at the surface of the mature Spike trimer and in particular the residues that form the loops may be accessible for antibody binding. We did not find direct evidence in the literature that antibodies against the sequence covered by S-657 can neutralize infection. Intriguingly, however, one report demonstrated *in vitro* that patient plasma with high levels of antibodies against the region 655-672 inhibited furin proteolysis (27). This report and the fact that antibody Fc-mediated effector functions are likely to contribute to protection from SARS-CoV-2 infection as well (28, 29) support the notion that vaccine-elicited antibodies to S-657 might be functionally relevant.

We observed with S-657 an epitope that distinguished the NVX-CoV2373 protein vaccinees from the other two vaccine cohorts. This raises the question of whether this was merely circumstantial or whether it was based on molecular or immunological reasons. Any aspect related to the presentation of the antigen to the immune system that differs between vaccines could have an impact. On the sequence level of the presented Spike protein, NVX-CoV2373 has a crucial difference to AZD1222 and BNT162b2 in that it carries the 3Q replacement (R682, R683, and R685 switched to Q) that inactivates the furin cleavage site. The rationale for this sequence change was to maximally stabilize the pre-fusion conformation of Spike to raise potent neutralizing antibodies (30). The direct structural impact of the 3Q exchange on the S1 SD2 domain immediately preceding the furin cleavage site remains unclear. The reason is that the available 3D structures of Spike do not resolve the furin cleavage site, which is also the case for the 3Q structure (31). However, since S-657 is just about 30 amino acids N-terminal to the furin cleavage site in SD2, it is tempting to speculate that the 3Q stabilization leads to a different presentation of Spike to the immune system and in effect a lower probability to raise antibodies against the S-657 sequence. With respect to the pathways of presentation to the humoral immune system, the NVX-CoV2373 protein vaccine is directly exposed together with an adjuvant to immune cells, e.g. dendritic cells for antigen presentation. In contrast, the adenoviral and mRNA vaccines are taken up by various cell types that eventually express and present Spike protein on their plasma membrane where B-cells can engage it [for review see (32)]. These differences might, therefore, also play a role in a different B-cell epitope pattern.

The sequences of S-813 and S-1145 stood out as targets of our patient cohort in comparison to pre-COVID and vaccinee samples. S-813 overlaps with a region known as the ‘fusion peptide that follows the S2’ cleavage site at residue R815. It is mechanistically involved in SARS-CoV-2 entry into the host cell (21). The reactivity of antibodies in the blood of COVID-19 patients and convalescents towards this part of Spike S2 has been described numerous times (11–14, 33–37). Importantly, it has been shown that patients´ antibodies that target the fusion peptide can have robust virus-neutralizing activity (38, 39). Peptide S-1145, on the other hand, resides in an alpha helix of the S2 stem part of Spike in the “connector domain”. Sequences that overlap with this peptide are already known targets of antibodies from SARS-CoV-2-infected people (11–14, 33–37). Patient-derived monoclonal antibodies targeting such epitopes neutralize viral infection, probably by inhibiting conformational changes of Spike required for membrane fusion, but also by Fc-dependent antibody effector activities (40, 41). The importance of antibodies against sequences covered by S-813 and S-1145 has been reinforced in a recent publication: Both sequence regions consist of evolutionarily conserved residues (named “coldspots”) and elicit neutralizing antibodies that cross-react not only with the Spike protein of all tested SARS-CoV-2 variants but also Spike from many other coronaviruses (42). Optimized vaccine design and informed selection of therapeutic antibodies will therefore provide new opportunities to counteract viral evolution.

Our study has some limitations, among them the investigation of only linear 15mer peptides that were attached to a matrix *via* their N-terminus. While even small peptides can adopt secondary structures that are recognized by the humoral immune response, larger portions or the whole Spike protein fold into 3D conformations that also present discontinuous epitopes. Such assembled epitopes are known to be the target of neutralizing antibodies, in particular within the RBD region of Spike S1 (43). We have not investigated here whether any of the linear peptide-specific antibodies that we have discovered in patient and vaccine plasma have a neutralizing capacity, and thus we can only speculate on their significance. On the other hand, S-657 signals correlated well with anti-RBD titers obtained *via* a standard diagnostic immunoassay. The S-657 antibodies may therefore be part of a larger humoral immune signature targeting immune dominant epitopes on Spike (11, 44–46).

Another limitation lies within our experimental setup, which consisted of initial screening with plasma pools obtained from either SARS-CoV-2 patients with active disease or AZD1222 and BNT162b2 vaccinated groups at six months after initial immunizations. Subsequent analyses of plasma from NVX-CoV2373 vaccinees *via* ELISA, therefore, prevented the identification of novel peptides not previously recognized by AZD1222 or BNT162b2 vaccinees. Likewise, antibodies that developed at later time points yet were specific for additional peptides could not be picked up by our ELISA screen. It is therefore possible that we missed epitopes. Moreover, our results may have experienced some skewing by the fact that the patient´s plasma had been collected during the first two weeks after infection (15), while the vaccinees´ plasma was obtained 6 months after initial vaccination (47). Indeed we could show in a previous study that a heterologous prime-boost with AZD1222 and BNT162b2 increased overall antibody levels and the neutralization capacity of plasma samples from vaccinated persons (47). Such observations support a scenario for novel epitopes to emerge upon heterologous vaccination.

In conclusion, our study contributed comparative landscapes of linear B-cell epitopes for three vaccine cohorts, among them NVX-CoV2373 vaccinees. We identified with S-657 an immunodominant region within subdomain 2 of the Spike S1 part that elicited higher immune reactivity after vaccination with the nucleic acid-based vaccines than with NVX-CoV2373. This sequence region is so far unaltered in viral variants and, based on literature information, a target of antibodies that may inhibit virus function. Understanding the epitope patterns is essential to optimize vaccine design, diagnostic assay development, and even therapy, e.g. generation and use of antibodies as drugs. In particular, the knowledge of immunodominant and immutable targets on Spike protein will be instrumental to designing vaccines and therapeutic antibodies that likely do not lose efficacy in circulating SARS-CoV-2 variants or those to come.

## Data availability statement

The original contributions presented in the study are included in the article/**Supplementary Material**. Further inquiries can be directed to the corresponding author.

## Ethics statement

The studies involving human participants were reviewed and approved by Ethics Committee of the University Medical Center of Rostock (reference number A 2020-0086, date of approval: 16 June 2021). The patients/participants provided their written informed consent to participate in this study.

## Author contributions

Conceptualization, PL and BM-H; methodology, PL, FS and FM; validation, PL and FS; formal analysis, PL, FS, and BM-H; data curation, PL, FS, and BM-H; writing—original draft preparation, PL and BM-H; writing—review and editing, PL, FS, FM, ER, and BM-H; visualization, PL and FS; supervision, BM-H; funding acquisition, ER. All authors have read and agreed to the published version of the manuscript
