# Supplementary material for: A linear B-cell epitope close to the furin cleavage site within the S1 domain of SARS-CoV-2 Spike protein discriminates the humoral immune response of nucleic acid- and protein-based vaccine cohorts
Source: Front Immunol. 2023 May 5;14:1192395. doi: 10.3389/fimmu.2023.1192395 (PMC10203960; doi:10.3389/fimmu.2023.1192395)
Supplement: Supplementary file 1 [file DataSheet_1.pdf]

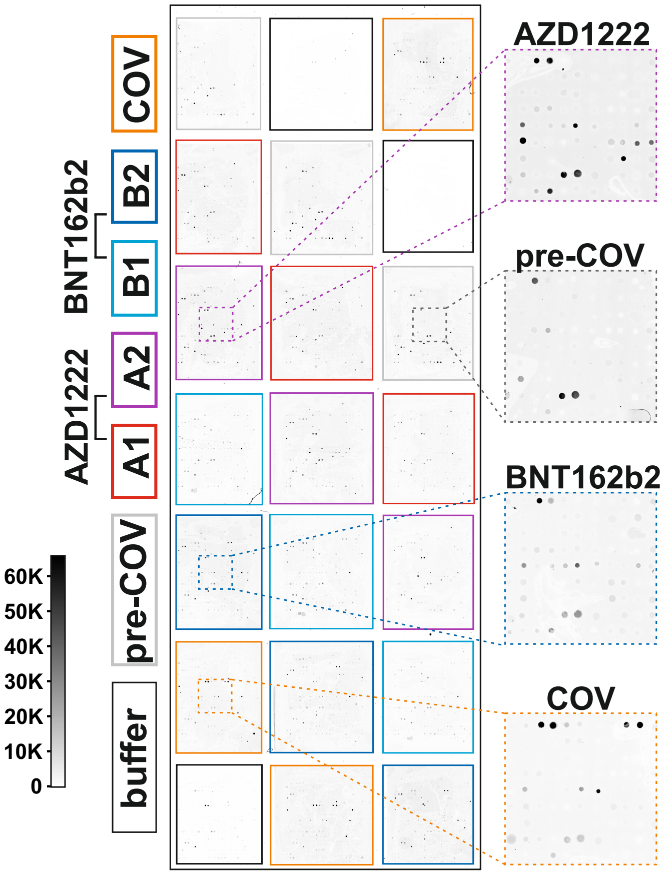

Supplementary Figure S1. Visual representation of a slide carrying 21 peptide microarrays. Rectangles with identical color frames indicate replicas. Sample pools according to legend to the left included two vaccinee pools each, for AZD1222 and BNT162b2, one plasma pool for COVID-19 patients with active disease (COV) and one from pre-pandemic donors (pre-COV). The label "buffer" indicates background controls that did not receive any plasma sample. Magnified details on the right show microarray regions of individual samples, one for each group.
