# Supplementary material for: A linear B-cell epitope close to the furin cleavage site within the S1 domain of SARS-CoV-2 Spike protein discriminates the humoral immune response of nucleic acid- and protein-based vaccine cohorts
Source: Front Immunol. 2023 May 5;14:1192395. doi: 10.3389/fimmu.2023.1192395 (PMC10203960; doi:10.3389/fimmu.2023.1192395)
Supplement: Supplementary file 2 [file DataSheet_2.pdf]

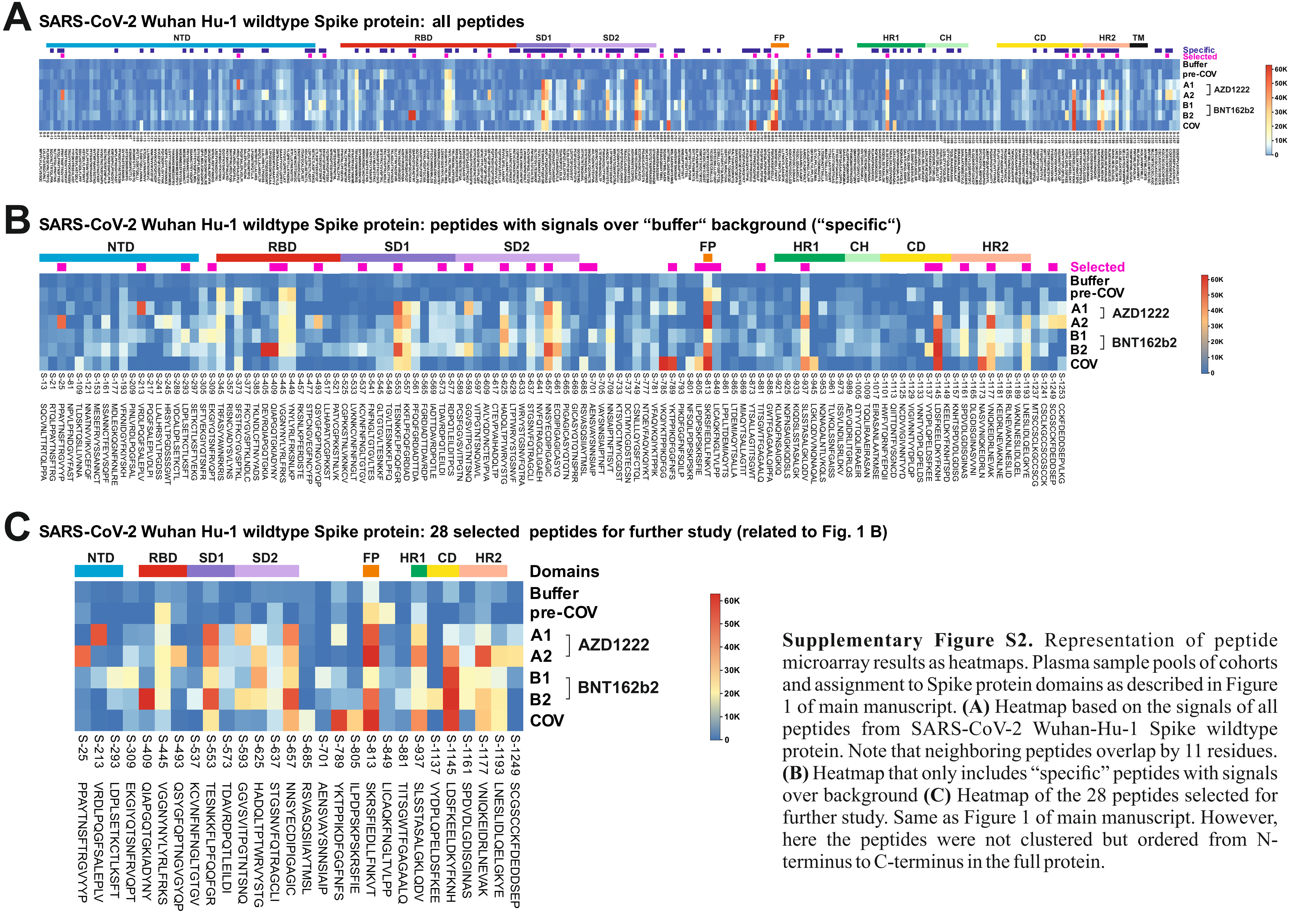

Supplementary Figure S2. Representation of peptide microarray results as heatmaps. Plasma sample pools of cohorts and assignment to Spike protein domains as described in Figure 1 of main manuscript. **(A)** Heatmap based on the signals of all peptides from SARS-CoV-2 Wuhan-Hu-1 Spike wildtype protein. Note that neighboring peptides overlap by 11 residues. **(B)** Heatmap that only includes “specific” peptides with signals over background **(C)** Heatmap of the 28 peptides selected for further study. Same as Figure 1 of main manuscript. However, here the peptides were not clustered but ordered from N-terminus to C-terminus in the full protein.
