# Supplementary material for: A linear B-cell epitope close to the furin cleavage site within the S1 domain of SARS-CoV-2 Spike protein discriminates the humoral immune response of nucleic acid- and protein-based vaccine cohorts
Source: Front Immunol. 2023 May 5;14:1192395. doi: 10.3389/fimmu.2023.1192395 (PMC10203960; doi:10.3389/fimmu.2023.1192395)
Supplement: Supplementary file 3 [file DataSheet_3.pdf]

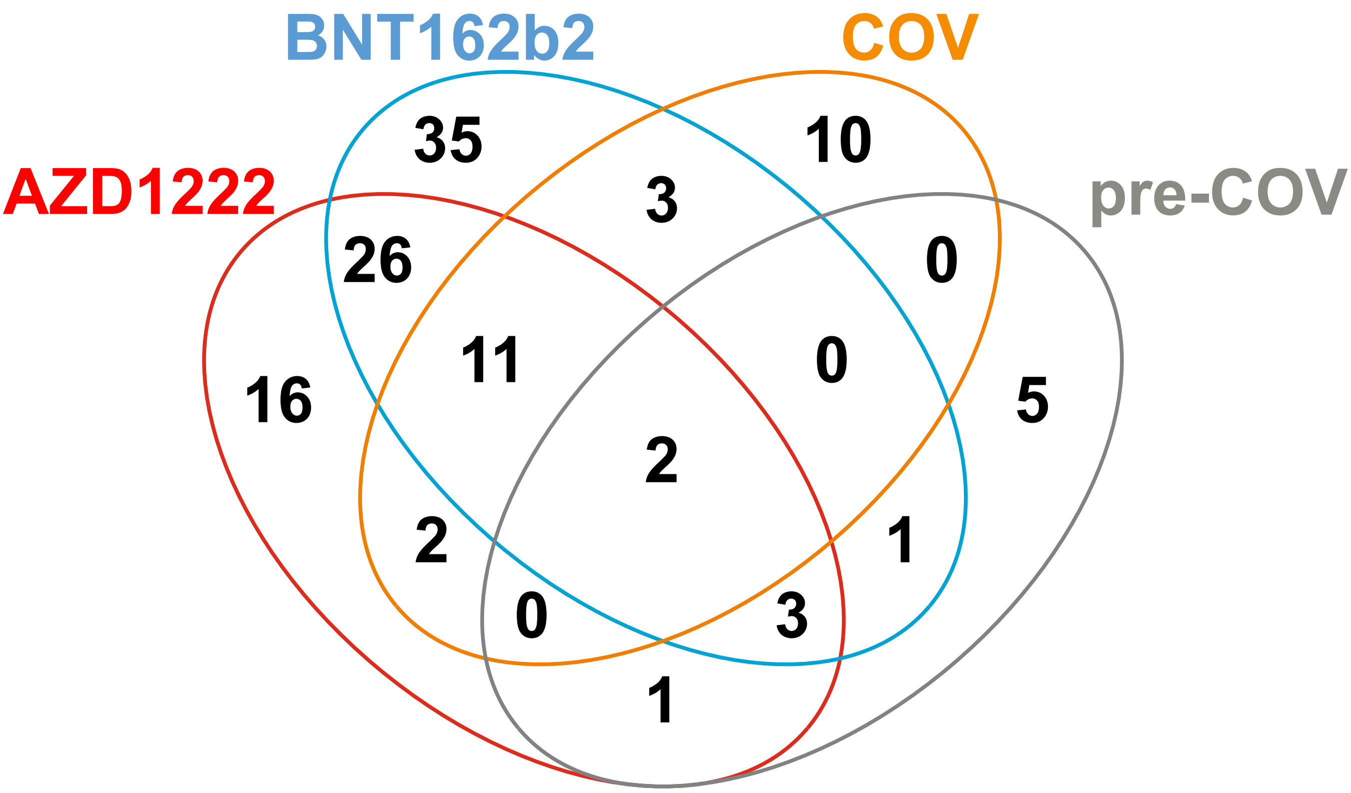

Supplementary Figure S3. Counts of shared and distinct reactive peptides for the peptide microarray measurements of the plasma pools representing AZD1222 and BNT162b2 vaccine cohorts, COVID-19 patients (COV) and pre-pandemic samples (pre-COV). Venn diagram (made with jvenn; http://jvenn.toulouse.inra.fr) based on the input of all reactive peptides derived from Spike Wuhan-Hu-1 wildtype protein.
